# Methodology for Modeling the Microbial Contamination of Air Filters

**DOI:** 10.1371/journal.pone.0088514

**Published:** 2014-02-11

**Authors:** Yun Haeng Joe, Ki Young Yoon, Jungho Hwang

**Affiliations:** 1 School of Mechanical Engineering, Yonsei University, Seoul, Republic of Korea; 2 Exhaust Emission Engineering Team, Hyundai Motor Company, Hwaseong, Republic of Korea; Ecole des Mines d'Alès, France

## Abstract

In this paper, we propose a theoretical model to simulate microbial growth on contaminated air filters and entrainment of bioaerosols from the filters to an indoor environment. Air filter filtration and antimicrobial efficiencies, and effects of dust particles on these efficiencies, were evaluated. The number of bioaerosols downstream of the filter could be characterized according to three phases: initial, transitional, and stationary. In the initial phase, the number was determined by filtration efficiency, the concentration of dust particles entering the filter, and the flow rate. During the transitional phase, the number of bioaerosols gradually increased up to the stationary phase, at which point no further increase was observed. The antimicrobial efficiency and flow rate were the dominant parameters affecting the number of bioaerosols downstream of the filter in the transitional and stationary phase, respectively. It was found that the nutrient fraction of dust particles entering the filter caused a significant change in the number of bioaerosols in both the transitional and stationary phases. The proposed model would be a solution for predicting the air filter life cycle in terms of microbiological activity by simulating the microbial contamination of the filter.

## Introduction

Bioaerosols are airborne particles of biological origins, which include viruses, bacteria, fungi, and all varieties of living materials [1]. In suitable hosts, bioaerosols are capable of causing acute and chronic diseases, which may be infectious, allergenic, or toxigenic [2], [3]. In order to control bioaerosols, numerous engineering solutions are commercially available or under development, including air filtration, ultraviolet germicidal irradiation (UVGI), air ionization, dielectric barrier discharge, and others [4]–[10]. Indoor bioaerosols accumulate in large quantities on filters of heating, ventilating, and air-conditioning (HVAC) systems, where they are able to multiply under certain conditions, especially if high amounts of moisture are present on the filters [11]–[13]. Moreover, the organic or inorganic materials deposited on the filter media following air filtration contribute to microbial growth. This inevitably leads to a decrease in filter efficacy and likely deterioration of the filters, with the eventual release of microorganisms. Microbial volatile organic compounds (MVOCs) produced by microbial metabolism can also be emitted from the contaminated filters [14].

Antimicrobial treatments are a possible solution to the aforementioned problems. Various antimicrobial agents, including iodine and silver, have been used in the treatment of air filters [15]–[16]. While antimicrobial treatments can delay the onset of bioaerosol entrainment, it does not completely prevent the release of microorganisms from contaminated air filters [17]. Such a result implies that microorganisms can grow on antimicrobial air filters, and the colonized antimicrobial air filters can be a source of bioaerosols if used over an extended period of time without replacement.

Dust particles decrease porosity of the filter media and interrupt airflow inside the filter. Consequently, the filtration efficiency of the air filter increases with the amount of dust particles deposited on the filter surface. Furthermore, dust particles can decrease the antimicrobial ability of a filter by preventing contact between the antimicrobial agent coated on the surface of the filter and the microorganisms. According to a series of qualitative experiments detailed in a report by the American Society of Heating, Refrigerating, and Air-Conditioning Engineers (ASHRAE), dust-loaded and non-dust-loaded panel filter pairs with different antimicrobial agents produced different results in microbial growth tests [18].

Based on the above discussion, the proper life cycle of antimicrobial air filters must be determined in order to maintain biologically clean and safe environment. To date, there has been no reliable and available technique for monitoring microbial contamination *in situ* according to a drop in pressure, which is an indicator of the final life cycle for general air filters. By developing a methodology for modeling microbial contamination of antimicrobial air filters and the entrainment of bioaerosols from contaminated filters, the life cycle of antimicrobial air filters could be predicted from a microbiological point of view.

A number of mathematical models for expressing of microbial growth in food and culture media have been developed [19]–[22]. The growth of organisms is often effectively described with the logistic model [23]. The logistic model, which represents microbial growth, is based on a differential equation with the following form:
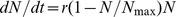
(1)where 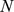 is the population (arithmetic number) of the organism at time *t*, 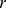 is the growth rate, and 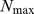
is the maximum population (at the stationary phase), often referred to as the carrying capacity of the environment. Here, 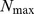
is an asymptote; 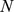 can be very close, but not equal to 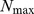. The logistic model contains the term 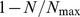, which suppresses the growth rate when the population is high. When 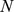 is very small, the value of this term is almost one and thus, it does not affect the growth rate. As 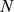 increases so as to be close to 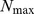, the value of 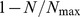 approaches zero, thus making the rate of growth almost zero [24].

In this study, a simple numerical method to model both microbial growth on an antimicrobial air filter and the entrainment of bioaerosols from the filter to the indoor environment is proposed by modifying the conventional logistic model. The suggested method considers the penetration of bioaerosols, filtration, and antimicrobial action of the antimicrobial air filter. The growth rate and entrainment rate are computed by comparing them with the respective rates obtained in previous experimental studies. In addition, the effects of deposited dust particles on filtration and antimicrobial action are investigated.

## Methods

### Model development

In general, the performance of an antimicrobial air filter is described in terms of the filter's filtration and antimicrobial efficiencies, which are in turn determined by microscopic structure parameters, including the fiber diameter, solidity, antimicrobial agents, and their concentrations. For convenience, we first focus our modeling on the filtration and antimicrobial efficiencies of the filters while ignoring their microscopic structures.

When bioaerosols of number flux, 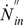 (CFU/cm^2^/hour), flow into an antimicrobial air filter, the deposited (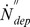, CFU/cm^2^/hour) and penetrated (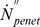, CFU/cm^2^/hour) fluxes of the bioaerosols are as follows:
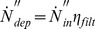
(2)

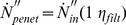
(3)where 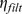 is the filtration efficiency of the antimicrobial air filter. Among the deposited bioaerosols, a fraction would be killed (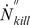, CFU/cm^2^/hour) by a reaction with the antimicrobial agent contained on the filter, while others would survive (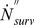, CFU/cm^2^/hour); these fractions can be expressed as follows:
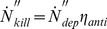
(4)

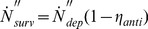
(5)where 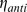 is the antimicrobial efficiency of the antimicrobial air filter. The deposited and surviving microorganisms would grow on the filter media and inevitably contribute to the microbial contamination of the filter. The increasing rate of microorganisms that exist on the filter over a time period *dt* can be expressed as follows:
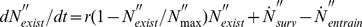
(6)where 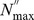 (CFU/cm^2^) is the maximum number of microorganisms that can exist on a unit area (1 cm^2^) of the filter, and 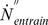 (CFU/cm^2^/hour) is the flux of bioaerosols entrained from the filter. In this study, it was assumed that 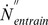 was proportional to the total amount of microorganisms existing in the filter by the entrainment rate, 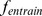 (1/hour), which was defined as the product of the entrainment constant (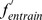, 1/m) and media velocity (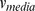, m/hour).
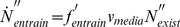
(7)

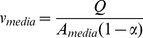
(8)where 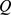 is the flow rate, 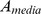 is the media area of the filter, and 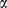 is the solidity of the filter.

The numerical solution of Eq. 6 at a certain time *i* was calculated with the first backward discretization method as follows:
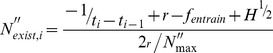
(9)

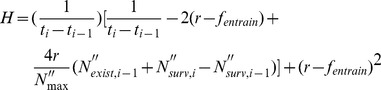
(10)


The number of bioaerosols observed downstream of an antimicrobial air filter (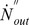, CFU/cm^2^/hour) is the sum of bioaerosols that penetrated through the filter and entrained from the filter.
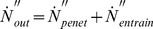
(11)


The solidity of the filter increases when the dust particles of mass flux (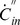, g/cm^2^/hour) are deposited on the filter surface, which leads to an increase in the filtration efficiency and media velocity. Among several mathematical models used to predict the filtration efficiency with a variation in solidity, the Rubow model [25] was selected for this study [26]. The filtration efficiency for any particle size and set of conditions can be calculated as follows [27]

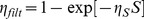
(12)where 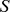 is the dimensionless fiber projected area. The mathematical definition of the dimensionless fiber-projected area is as follows [27]–[28]:
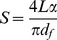
(13)where 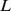 is the length of the filter media in the direction of air flow and 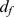 is the fiber diameter. In Eq. 12, 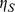 is the single-fiber efficiency, the values of which depends on particles size, air velocity, and fiber properties. The single-fiber efficiency can be represented as the sum of the single-fiber efficiencies by diffusion (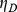) and interception (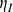), which are expressed as follows:
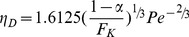
(14)

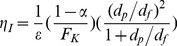
(15)where 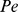 is the Péclet number, 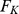 is the Kuwabura hydrodynamic factor, and 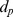 is the particle diameter. The correction factor 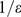 accounts for filter media inhomogeneities, and the value of 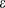 is approximately 1.6 for glass fiber filters. For details, readers are referred to research by Kowalski et al. [26].

To evaluate the effect of deposited dust particles on filter solidity, the following equation is considered:
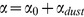
(16)where 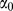 is the filter solidity without dust loading, and 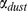 is the solidity added by the deposited dust particles, which can be calculated according to the following equation:
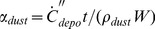
(17)where 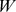 is the thickness of the filter media. Here, the dust particle density (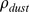) is assumed to be the density of the silica particle, 2.7 g/cc. The dust deposition mass flux onto the filter, 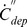 (g/cm^2^/hour), can be calculated with the following expression:
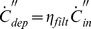
(18)


In our previous study, the effect of the dust loading amount on antimicrobial characteristics of an antimicrobial air filter was investigated [29]. Silver nanoparticles, which have a mode diameter of 16 nm, were used as the antimicrobial agent, and two types of bacteria, *Staphylococcus epidermidis* and *Escherichia coli*, were employed. The antimicrobial ability of the silver nanoparticle-coated air filter linearly decreased as the amount of dust particles increased. Therefore, in this work, we assumed that the antimicrobial efficiency of an antimicrobial air filter linearly decreased with the amount of dust particles deposited on the filter:
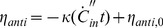
(19)where 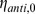 is the antimicrobial efficiency when dust effects are absent. The coefficient 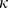 represents the sensitivity of the antimicrobial ability against dust loading. Here, 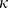 was set as 60 cm^2^/g [29].

Nutrient components, which are included in the dust particles, can affect both the growth rate (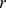) and the maximum number of microorganisms (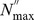) on the filter. In research by Fujikawa and Morozumi, the effects of nutrient level on the growth rate of bacteria and the maximum number of bacteria on the surface of a membrane filter (diameter 25 mm) were investigated [30]. By plotting their experimental results with SigmaPlot 8.0 (SPSS Inc.) commercial software, the growth rate and maximum number of microorganisms were modeled as a function of the amount of deposited dust particles as follows: 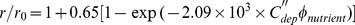
(20)

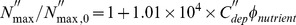
(21)where 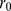 and 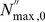 are the growth rate and maximum number of microorganisms when no dust effects are considered, respectively, and 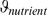 is the weight fraction of nutrient components in the dust particles. The expressions of 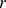 and 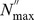 were in good agreement with previous experimental results, showing *R* ^2^ of 0.93, and 0.99, respectively.

### Test particle size distributions

To determine dust and bioaerosol particle filtration efficiencies, the sizes of the particles should be known. Size distributions of ambient particles are commonly represented in nuclei, accumulation, and coarse particle modes along with their sources, size ranges, formation mechanisms, and chemical compositions [31]. Each mode can be described by a log-normal distribution; the result reported by Whitby [32] was used as the model distribution of dust particles in this study. Moreover, experimental data obtained by Górny et al. were utilized to determine the log-normal distribution of bioaerosols [33]. The parameters for each model distribution employed in this study are summarized in Table 1.

**Table 1:** Parameters of model particle distributions.

Contents		Mass median diameter (µm)	Geometric standard deviation	Fraction
Dust particle	Nuclei mode	0.039	1.8	0.768
	Accumulation mode	0.320	2.16	0.231
	Coarse particle mode	5.673	2.21	0.001
Bioaerosol particle		5	1.8	1

### Determination of parameters

Several parameters were determined without considering dust particles. First, the maximum population of microorganisms on the filter without dust effect (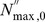) was determined according to results obtained in previous work [30], where 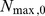 of *E. coli* was 10^7.75^ CFU (colony forming units) on the surface of a membrane filter (diameter 25 mm). Therefore, 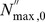 was assumed to be 1.15×10^7^ CFU/cm^2^.

The growth rate (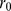) and entrainment constant (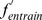) were then determined by referencing another previous report [17], in which 50 µL of a mixed culture (6×10^8^ cells/mL) consisting of ten bacterial and six fungal species was inoculated into each filter specimen (diameter  = 4.8 cm) of a high-efficiency particulate air (HEPA) filter composed of microfiber glass and acrylic resins. Three types of filter specimen were used; a HEPA filter treated with antimicrobial agent A, a HEPA filter treated with antimicrobial agent B, and a normal HEPA filter. All the specimens were incubated from seven days to three months at 25°C with a relative humidity higher than 90%. At ten-day intervals, the specimens were removed from the incubator. Each specimen was located in a test duct through which air was flowing. Bioaerosols entrained from the specimen were sampled using a sterile cellulosic membrane filter. The media velocity for the selection and sampling time were 0.02 m/sec and 5 min respectively. To simulate previously published results, the solution of Eq. 6, without considering the surviving bioaerosol (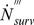) and entrained bioaerosol (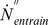) particles, is represented as follows [23],
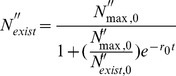
(22)where 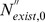 is the number concentration of bioaerosols that initially existed on a unit area of the filter. The number concentration of entrained bioaerosols per unit area of the filter can be obtained by integrating Eq. 7, assuming that the growth rate and the entrainment rate are constant.
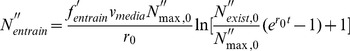
(23)


**Figure 1:**
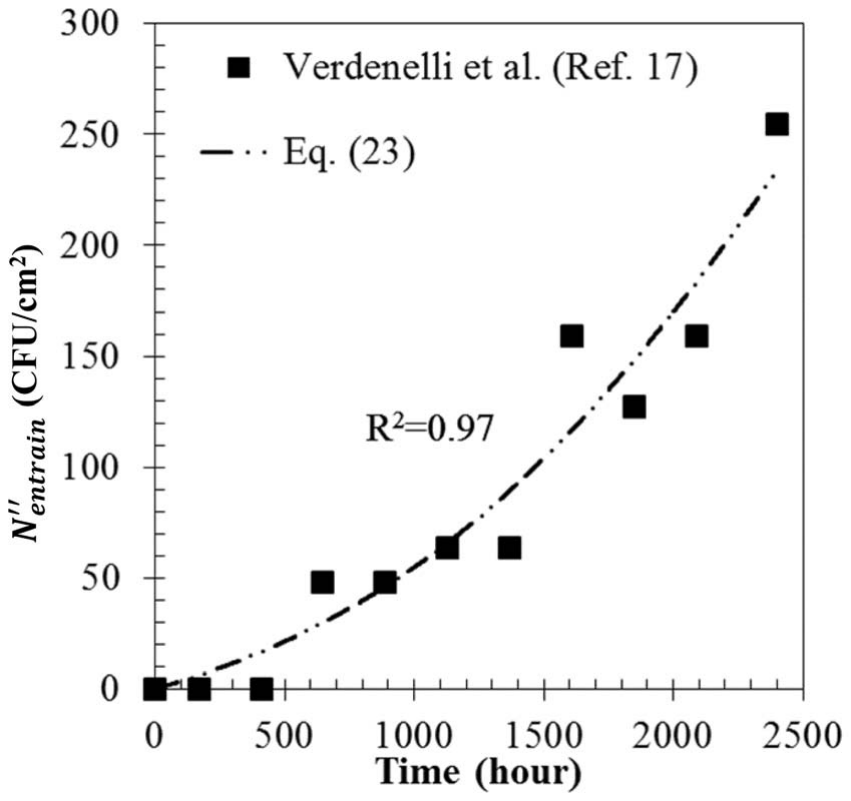
Determination of the growth rate and entrainment constant. Comparison of the calculation results (line) and the experimental finding (solid square).

By substituting 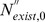 = 1.66×10^6^ CFU/cm^2^ [17] into Eq. 23 and comparing our calculated results with experimental data of a previous work of Verdenelli et al. [17], the growth rate (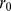) and the entrainment constant (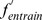) were determined with SigmaPlot 8.0. Finally, 0.0012 1/hour and 3.0×10^−10^ 1/m were selected as proper values for the growth rate and entrainment constant, respectively, showing *R* ^2^ of 0.97, as presented in Fig. 1.

In ASHRAE Standard 52.2–2007 [34], air filters are classified into 16 grades with respect to the minimum efficiency reporting value (MERV). In this study, four air filters with different filtration efficiencies (MERV 14, 11, 9, and 8) were selected. The filtration efficiency for each filter was calculated by the Rubow model [21] with Whitby particle distribution [32] at 1 m^3^/s of flow rate. The specifications of the model filters are summarized in Table 2. Moreover, three different antimicrobial efficiency (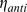) were applied to each model filter: 99% (most of captured bioaerosols are killed), 50% (half of captured bioaerosols are killed), and 0% (non-antimicrobial filter).

**Table 2:** Specifications of model filters.

Contents	Filter 1	Filter 2	Filter 3	Filter 4
Solidity ( 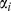 )	0.008	0.002	0.002	0.001
Media length ( 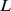 , m)	0.017	0.015	0.015	0.015
Face area (m^2^)	0.35	0.35	0.35	0.35
Media area (m^2^)	10	10	10	10
Fiber diameter ( 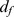 , µm)	3	3	4.5	4.5
Thickness ( 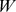 , mm)	25	25	25	25
MERV @ Q = 1.0 m^3^/s	14	11	9	8

Using the identified parameters, antimicrobial air filters were modeled as a function of filter operating time under a constant bacterial concentration (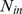), 500 CFU/m^3^, three constant dust concentrations (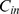) 0 µg/m^3^ (without dust), 100 µg/m^3^, and 500 µg/m^3^ and three constant flow rates (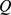) 0.5, 0.75, and 1.0 m^3^/sec (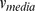 are 0.05, 0.075, and 0.1 m/s, respectively). Three constant weight fractions of nutrient components (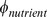), 0, 0.05, and 0.1 were used.

### Calculation algorithm

**Figure 2:**
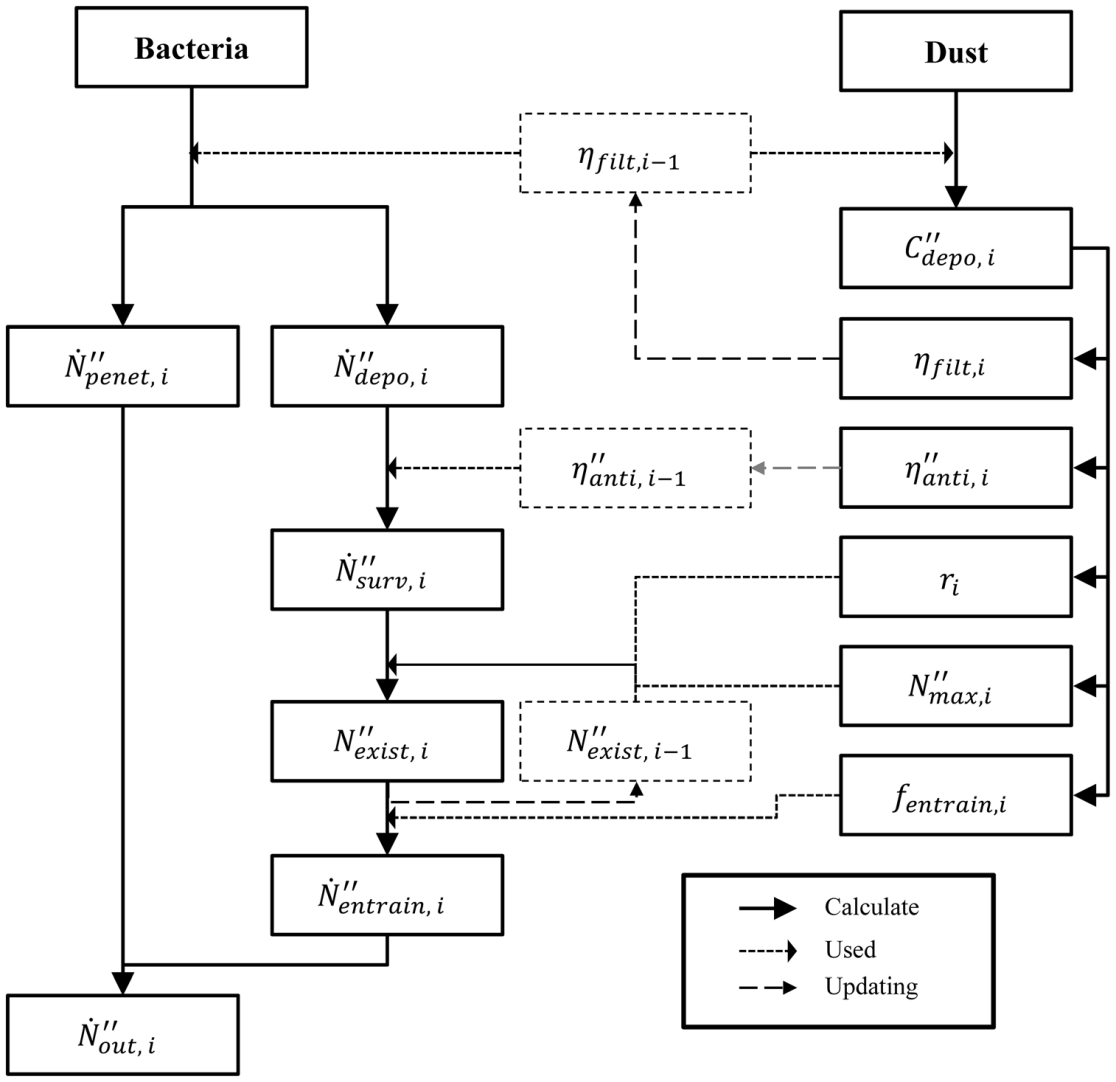
The calculation algorithm used to solve the equations simultaneously.

The calculation algorithm to solve the proposed equations is expressed in Fig. 2. In a certain operating time step (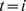), the amount of deposited dust (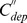), penetrated bioaerosols (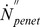), deposited bioaerosols (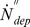), and surviving bioaersols (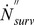) were calculated with filtration and antimicrobial efficiencies at the time step of 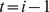. After the growth rate (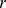) and entrainment rate (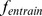), the maximum population of microorganisms on the filter (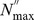) were updated by the calculated 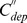 result. The existing (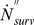) and entrained (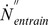) bioaerosols were calculated according to Eq. 9–10 and Eq. 7–8, respectively. Finally, 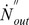, which is the sum of the penetrated and entrained bioaerosols, was calculated. In order to compute the next time step, 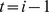, the filtration efficiency and antimicrobial efficiency were updated by the value of 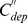 determined at 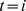. This full set of steps was then repeated. In order to convert the data per hour to data per day, a summation of data per hour over one day was carried out sequentially.

## Results and Discussion

### General characteristics

**Figure 3:**
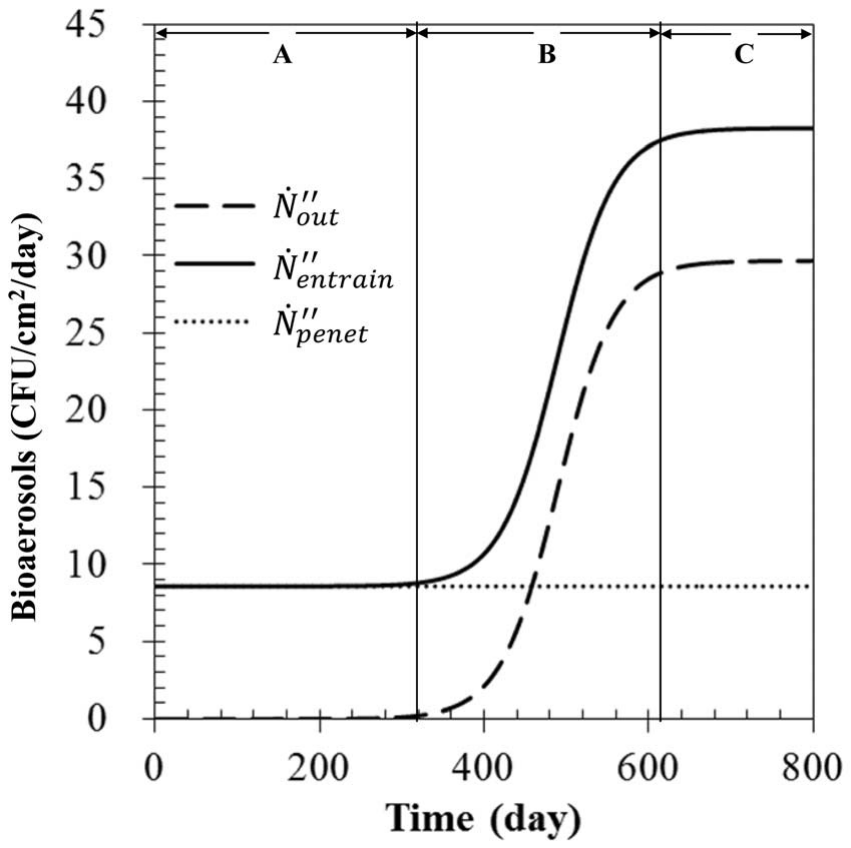
Characteristics of N˝•_entrain_, N˝•_penet_, and N˝•_out_ of Filter 2 as a function of filter operating time. 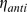
 = 0.5, 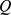 = 1.0 m^3^/sec, and 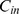 = 0 µg/m^3^.

Figure 3 illustrates the temporal variations of 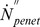, 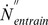, and 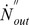 (defined as the sum of 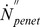 and 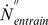) of Filter 2 with 50% of antimicrobial efficiency. The air flow rate was 1 m^3^/sec, and dust particles were not considered. Because Filter 2 had the constant filtration efficiency (MERV 11, see Table 2), 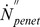 was also constant. The results show that the state of 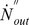 was divided into three phases: initial (A), transitional (B), and stationary (C). In the initial phase, 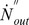 was almost the same as 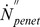, which was affected only by filtration efficiency. During this phase, 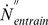, which is proportional to 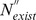 (see Eq. 7), was almost zero because 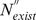 on the filter was not substantial enough to cause any entrainment of bioaerosols into the air stream. After approximately 370 days of use, an entrained bioaerosol was observed (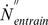
>1 CFU/cm^2^/day), and 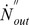 began to increase thereafter. At this transitional phase, 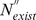 on the filter was sufficiently increased so as to cause entrainment. Such an increase continued until 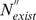 approached 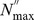. In the stationary phase, 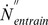 and 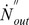 stopped increasing because 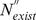 almost reached 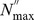.

### Effects of filtration and antimicrobial efficiencies

**Figure 4:**
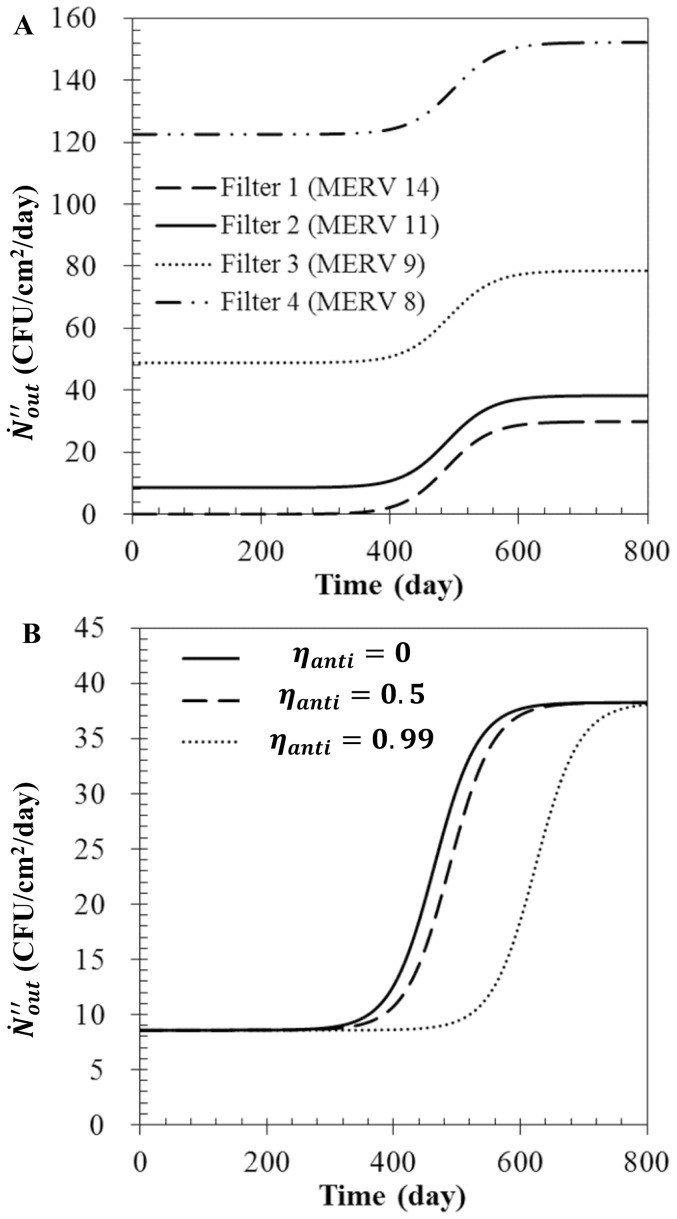
The N˝•_entrain_ Characteristics of air filters. (A) Various filter types when 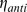 = 0.5, and (B) various antimicrobial efficiencies of Filter 2 with 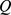 = 1.0 m^3^/sec, and 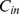 = 0 µg/m^3^.

The effect of the filtration and antimicrobial efficiencies on 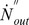 are displayed in Figures 4A and 4B, respectively; the air flow rate was 1 m^3^/sec, and dust particles were not considered. Fig. 4A shows 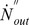 for four different model filters when 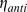 = 0.5. Higher filtration efficiency (Filter 1>Filter 2>Filter 3>Filter 4) led to a reduced penetration efficiency and thus, a lower value of 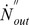 was observed. For higher filtration efficiency, the transitional phase started slightly earlier because a larger quantity of bioaerosols accumulated, survived, and multiplied on the air filter. Fig. 4B shows 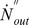 from Filter 2 with various antimicrobial efficiencies. Under the condition of constant filtration efficiency, the starting time of the transitional phase (i.e., when the microorganisms deposited on the filter began to release) was delayed with the increase in antimicrobial efficiency. However, even with the higher antimicrobial efficiency filter, 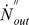 rapidly approached a maximum value once the operating time was in the transitional phase.

### Effect of dust particles

**Figure 5:**
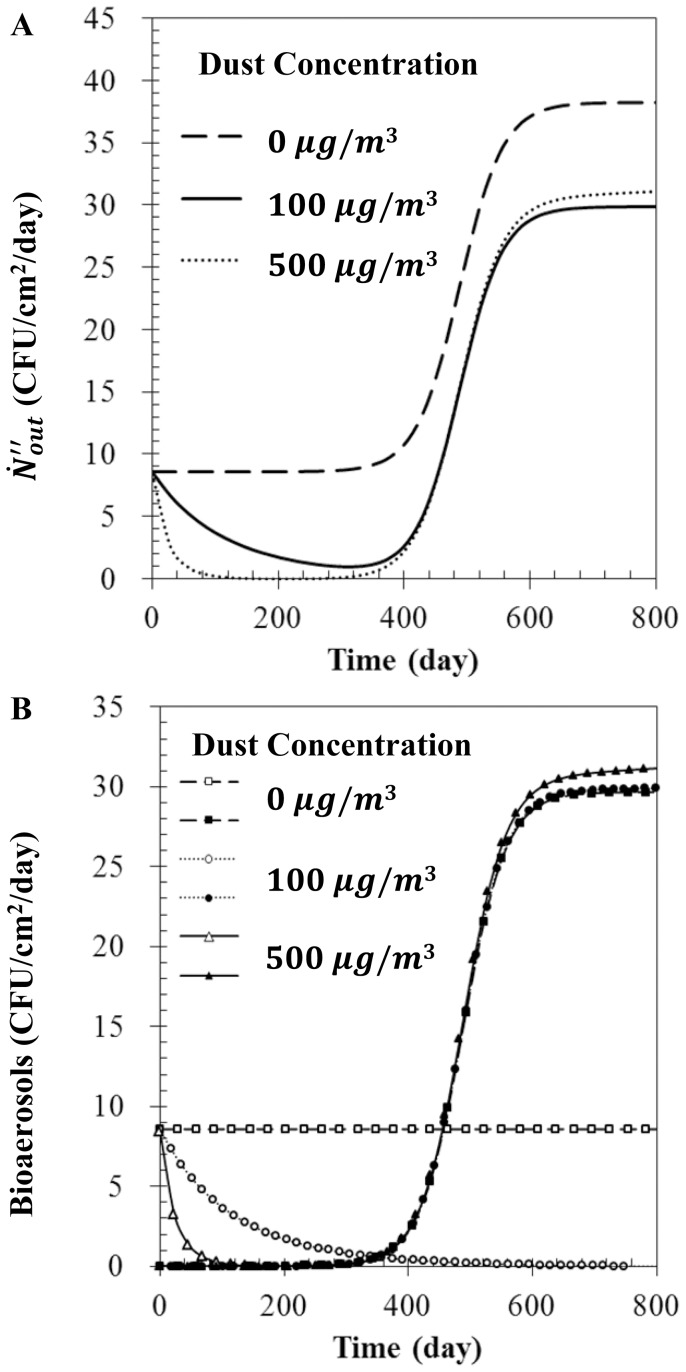
Bioaerosol number flux with various dust concentrations. (A) 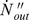, (B) 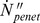 (open symbol), and 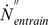 (filled symbol) for Filter 2 when η_anti_ = 0.5, 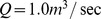, and 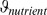 = 0.

The deposition of dust particles on the filter surface led to an increase in filter solidity. Furthermore, the filtration efficiency gradually increased with the solidity. For example, the result calculated from Eq. 12–18 show that the filtration efficiency for a dust concentration of 500 µg/m^3^ became 99.9% when the filter was used for 660 days. Consequently, the 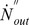 in the initial phase decreased with the amount of deposited dust particles, as shown in Fig. 5A.

The temporal variation of 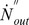 will now be discussed along with changes in 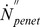 and 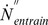 (recall 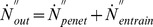). For any amount of dust, the continuous use of the filter caused entrainment of bioaerosols into the air stream, resulting in the increase of 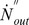 in the transitional phase. Fig. 5B shows that a higher concentration of dust particles caused a larger decrease in 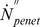 (open symbol), while no remarkable changes in 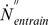 (filled symbol) was induced. Although the filter solidity increased with the deposition of dust particles, the media velocities with and without dust particles were almost same, resulting in negligible changes in the amount of entrained bioaerosols (see Eq. 7).

### Effect of flow rate

**Figure 6:**
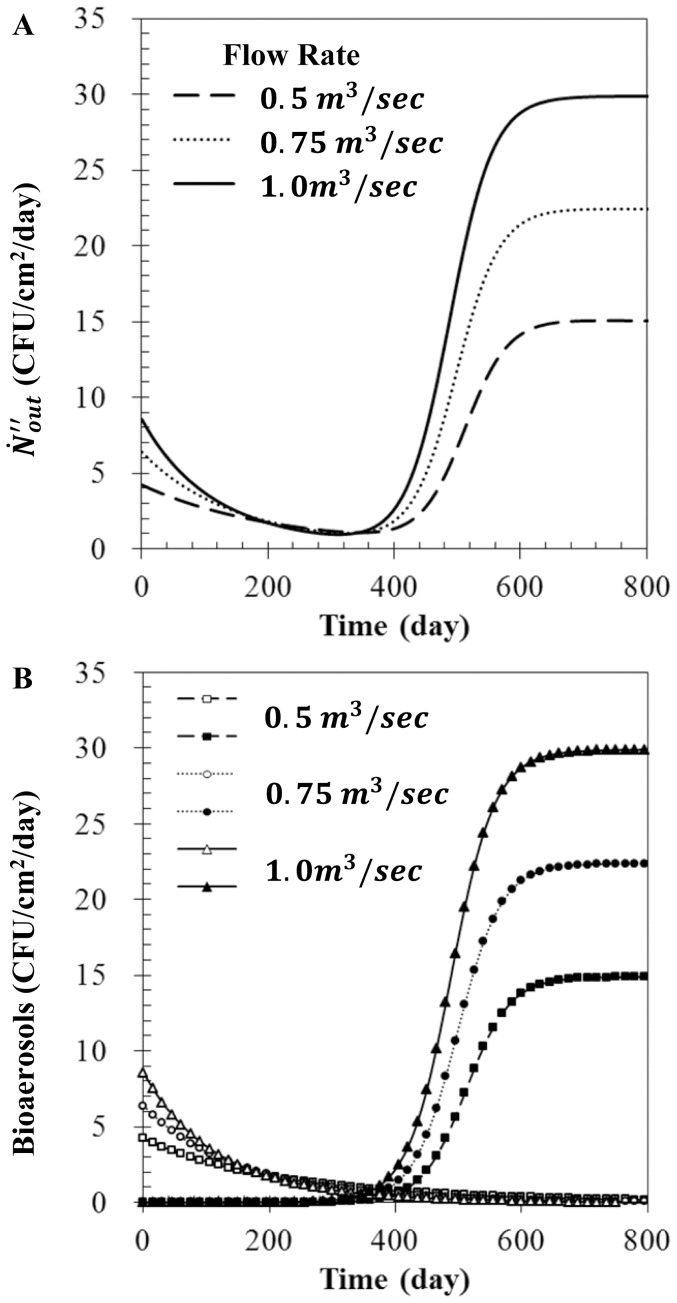
Bioaerosol number flux with various flow rates. (A) 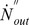, (B) 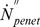 (open symbol), and 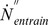 (filled symbol) for Filter 2 when 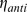 = 0.5, 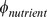 = 0, and 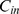 = 100 µg/m^3^.

An increase in the flow rate caused an increase in the entering bioaerosols and dust particles concentrations. The effect of flow rate on 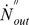 will now be discussed. The values of 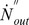 with various flow rates when η_anti_ = 0.5, 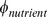 = 0, and 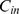 = 100 µg/m^3^ are displayed in Fig. 6A. The value of 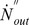 with a higher flow rate was larger than the value obtained at a lower flow rate. For any flow rate, 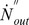 initially decreased, then increased with time before approaching a steady-state value. Fig. 6B shows that the initial amount of 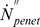 (open symbol) at a higher flow rate condition was larger than at the lower flow rate condition. However, for a higher flow rate, the increase in filtration by dust loading progressed more rapidly. Furthermore, at a higher flow rate, the starting time of the transitional phase was advanced, the value of 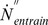 (filled symbol) increased more rapidly in the transitional phase, and a large amount of 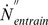 was observed in the stationary phase. In this paper, 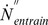 was modeled as a product of 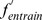, 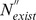, and 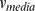 (see Eq. 7). The value of 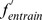 was assumed to be 3.0×10^−10^ 1/m. When the flow rate was increased from 0.5 m^3^/s to 1.0 m^3^/s, the media velocity increased from 0.05 to 0.1 m/s. However, our calculation shows that 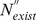 was nearly independent of flow rate. Consequently, the changes of 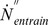 by flow rate were caused by changes of media velocity.

### Effect of nutrient level

**Figure 7:**
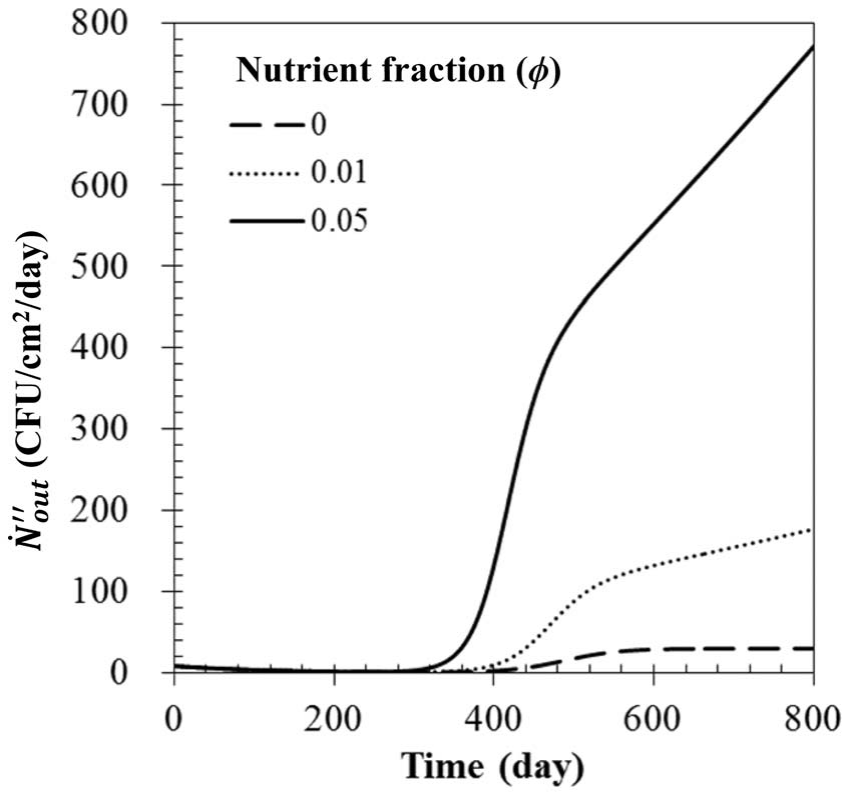
Bioaerosol number flux with various nutrient fractions. Here, 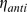 = 0.5, 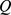 = 1.0 m^3^/sec, and 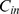 = 100 µg/m^3^.

Figure 7 shows the effect of nutrient fraction on 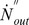 when 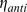 = 0.5, 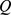 = 1.0/sec, 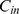 = 100 µg/m^3^. Because nutrient fraction affects the growth rate (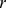) and the maximum number of bioaerosols (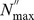) existing on the filter (See Eq. 20 and 21), there were critical differences in the transitional and stationary phases. With a rise in the nutrient fraction, 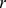 and 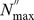 gradually increased, resulting in an increase of 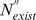 and 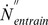. After 600 days of filter use, 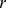 became 0.0016 1/hour and 0.0020 1/hour when the nutrient fractions were 0.01 and 0.05, respectively, while 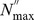 became 5.36×10^7^ CFU/cm^2^ and 2.22×10^8^ CFU/cm^2^. In the initial phase, 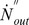 was almost equal to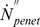, which was only affected by filtration efficiency.

### Effect of operation cycle

**Figure 8:**
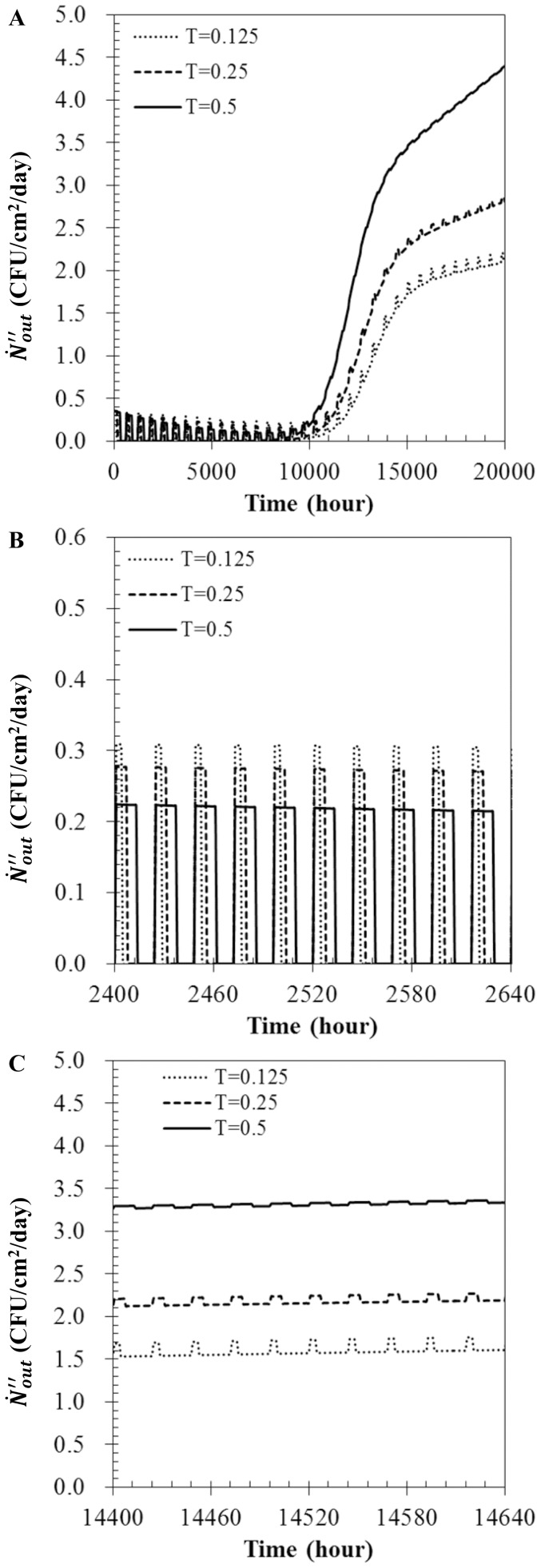
Bioaerosol number flux with various cycles of operation. (A) 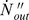, (B) details in initial phase (C) details in stationary phase when 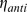 = 0.5, 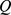 = 1.0 m^3^/sec, 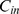 = 100 µg/m^3^, and 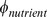 = 0.01.

In real-world applications, HVAC systems are turned on and off periodically. Thus, bioaerosols, dust particles, and nutrient components are intermittently supplied to the filter surface. Such non-continuous operation can lead to remarkable changes of growth, penetration, and entrainment of bacteria. In order to model this phenomenon, operation cycle of 3 (dimensionless period T = 0.125), 6 (T = 0.25), and 12 hours (T = 0.5) per day were considered. In Fig. 8A, many bumps in 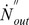 are observed, and the height of these bumps decreased with an increase in operating time. When the HVAC system was turned on, 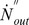 increased rapidly since the amounts of entered and penetrated bioaerosols increased. The amount of penetrated bioaerosols then became zero when the system was turned off. Details regarding the variations in the initial and stationary phases are shown in Fig. 8B and Fig. 8C, respectively. An increase in operating time served to increase the amount of dust particles deposited on the filter, which in turn caused an increase of the filtration efficiency and decrease in the amount of 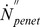. Furthermore, an increase of operating time increased the amount of nutrient component deposited on the filter, which caused an increase in both the growth rate (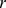) and the maximum number of bioaerosols (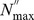) existing on the filter. Consequently, the starting time of the transitional phase was advanced and a large amount of 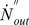 was observed at the stationary phase.

## Conclusions and Outlook

A modeling method for simulating the microbial contamination of antimicrobial air filters was proposed, and the number of bacteria downstream from the filter was calculated with variation of filtration and antimicrobial efficiencies, dust particle concentration, flow rate, and nutrient fraction of the dust particles. The characteristics of bioaerosols downstream of the filter were discussed with respect to three phases: initial, transitional, and stationary. Under the condition of constant filtration efficiency, the starting time of the transitional phase (i.e., when the microorganisms deposited on the filter began to release) was delayed with an increase in the antimicrobial efficiency. However, even with a higher antimicrobial efficiency filter, 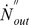 rapidly approached a maximum value once the operating time was in the transitional phase. In the case of filters with high filtration efficiency, bioaerosols were captured quite well. However, the large quantities of bioaerosols deposited on the filters accelerated the contamination process.

Dust particles led to increased filtration efficiency. While the filter solidity increased with the deposition of dust particles, the media velocities with and without dust particles were almost the same.

The initial amount of 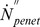 (open symbol) at a higher flow rate condition was larger than the amount in a lower flow rate condition. However, for a higher flow rate condition, the increase in filtration by dust loading progressed more rapidly. By increasing flow rate, the media velocity increased and thus, the starting time of the transitional phase was advanced, the value of 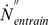 increased more rapidly in the transitional phase, and a large amount of 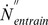 was observed in the stationary phase. The fraction of nutrients in the dust particles led to an increase in both the microorganism growth rate and the maximum number of bioaerosols that can exist on the filter.

Although our proposed model was useful for estimating the proper life cycle of an antimicrobial air filter, we recognize that the model includes empirical factors that limit the accuracy of the resulting estimates. First, the model is based on data that are insufficient for determining input parameters such as growth rate and entrainment constant. In the model, there parameters were ascertained using a single experimental work [17] under one environmental condition (25°C, RH >90%). Because microbial growth strongly depends on the environmental conditions (e.g., temperature and relative humidity) as well as the strain of bacterium, the effects of these parameters on the growth rate should be investigated [18], [24]. For these purposes, research into the selection of bacterial species which can represent indoor bioaerosols, as well as an evaluation of their growth rates on air filter media under various environmental conditions, must be performed. Secondly, in this study, 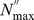 was assumed to be only a function of nutrient level on the filter surface. In reality, 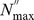 would vary for air filters with different filtration efficiencies because the filters have different surface characteristics (filter diameter, filter solidity, etc.) to support the microbial growth. An antimicrobial agent also can affect 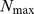 [35]. Lastly, the effect of dust particles on antimicrobial ability would vary with different antimicrobial agents and bacterial species.
